# Freeze–Thaw Durability of Strain-Hardening Cement-Based Composites under Combined Flexural Load and Chloride Environment

**DOI:** 10.3390/ma11091721

**Published:** 2018-09-14

**Authors:** Liqiang Yin, Changwang Yan, Shuguang Liu

**Affiliations:** 1 School of Materials Science and Engineering, Inner Mongolia University of Technology, Hohhot 010051, China; yinliqiang0720@126.com; 2 School of Mining and Technology, Inner Mongolia University of Technology, Hohhot 010051, China; ycw20031013@126.com

**Keywords:** strain-hardening cement-based composites (SHCC), freeze–thaw cycles, durability, flexural loading, chloride environment

## Abstract

Cement-based materials are usually not exposed to an independent deterioration process but are exposed to a combination of mechanical load and environmental effects. This paper reports the frost resistance durability of strain-hardening cement-based composites (SHCC) under combined flexural loading at different levels and under chloride attack. The loss of mass, dynamic elastic modulus, and microstructure characteristics of SHCC specimens were determined, and the influence of loading level on frost resistance was analyzed. In addition, the effect of freeze–thaw action on the flexural performance and diffusion properties of chloride in SHCC under the combined loads was investigated. The results show that the process of degradation was accelerated due to the simultaneous action of flexural loading and freeze–thaw cycles in the chloride environment, and SHCC suffered more serious damage at a higher loading level. However, flexural strength decreased by only 13.87% after 300 freeze–thaw cycles at load level S = 0.36. The diffusion properties of chloride in SHCC under constant flexural loading were affected by the freezing and thawing cycle. The free chloride concentration *C_f_* increased with the development of freezing and thawing at the same diffusion depth, and a bilinear relationship was found between the chloride diffusion coefficient *D*_c_ and the number of freeze–thaw cycles.

## 1. Introduction

Concrete is widely used in civil engineering, including for infrastructure and social facilities, as a safe and durable engineering material. In real field conditions, it is often subjected to tensile stress due to mechanical loads and environmental effects, and its brittle failure behavior is a particular concern [1]. Long-term durability is extremely important for all concrete structures, and it can be associated with the appearance of cracks when concrete is subjected to tensile stress. Once concrete cracks, crack width is difficult to control. The cracks can cause deterioration of concrete structures in a short period of time, and durability will be adversely affected [2]. Controlled crack width, delayed crack growth rate, and improved concrete toughness are key problems in improving the durability of reinforced concrete structures and in the sustainable development of infrastructure construction. In order to control the propagation of harmful cracks effectively, researchers have tried to add admixtures and fibers to concrete or improve the performance of concrete by controlling the molding process. Due to the lack of theoretical guidance and other reasons, improvement of concrete toughness is very limited.

Strain-hardening cement-based composites (SHCC), known as ultra-high-performance fiber-reinforced cementitious composites, was developed by Li [3]. SHCC was designed through the optimization of matrix, fiber, and interface properties, based on the theory of fracture mechanics and micromechanics. SHCC possesses a tensile strain capacity of more than 3% (300–500-fold higher than that of ordinary concrete) during the uniaxial tension phase duo to closely spaced multiple cracks with crack widths that are usually no more than 100 μm [4]. Under severe flexural loading, the deformation capacity of SHCC beams is similar to that of metal material [5]. In recent years, SHCC has rapidly become a concern of researchers around the world. Europe, America, Japan, China, Australia, South Africa, and other countries and regions have reported many experimental and theoretical research works on SHCC, which is considered one of the most promising materials in the field of civil engineering in the 21st century [6,7,8].

Much infrastructure located in cold regions is subjected to extremely harsh environmental conditions, where repeated freezing and thawing cycles and chloride erosion can seriously damage the integrity of a material or structure, causing rapid degradation of airfield runways, highway pavement, bridge deck slabs, and parking structures [9,10]. If SHCC is used for these types of structures, it must be resistant to freeze–thaw action. Preliminary tests of the frost resistance durability of SHCC indicated that it possesses superior freeze–thaw resistance [11]. The durability of SHCC subjected to mechanical loading and freeze–thaw action in a de-icing salt environment was reported in [12]. A visual rating of the surface and residual mass of SHCC remained within acceptable limits after 50 freeze–thaw cycles. Test results for reloaded specimens showed slight ductility loss, but they retained multiple crack behaviors and tensile strain capacity of more than 2.5–3%. In addition, it was also found that micro cracks induced by mechanical loading will heal automatically under freeze–thaw cycles in a salt solution, almost restoring the original stiffness. Experiments indicated that SHCC remained durable despite being exposed to freezing and thawing cycles in a de-icing salt environment [13]. It was found in [14] that frost resistance can decrease rapidly in a chloride environment compared to a freshwater environment, illustrating that a chloride environment has adverse effects on the frost resistance of SHCC. In addition, the microstructure characteristics of SHCC specimens before and after freeze–thaw cycles were described using scanning electron microscope images. After 300 freeze–thaw cycles, internal micro cracks were clearly observed [15]. The mechanical properties of SHCC were studied in [16] after various freeze–thaw cycle times. Test results showed that the action of 300 cycles had little effect on multiple micro-cracking behavior and deformability of SHCC specimens under uniaxial tensile and flexural loading. Studies have also shown that freeze–thaw action deteriorates the interface properties of the fiber-matrix and influences the strain hardening behavior of SHCC, eventually leading to decreased tensile strength and increased tensile strain capacity after freeze–thaw cycles [17].

Specifically, it was found that SHCC has superior anti-penetration ability, and chloride-proofing performance is affected by crack parameters such as the number of cracks, crack width, and spacing [18,19]. The chloride diffusion performance of SHCC specimens that were both cracked and uncracked, and the chloride contents were determined at different diffusion depths for the exposed surface of SHCC specimens was reported in [20,21]. Test results showed that cracks induced by loading in SHCC specimens lead to a higher rate of chloride migration than in uncracked SHCC. An experimental study on the durability of reinforced SHCC beam subjected to accelerated corrosion was conducted in [22]. Crack parameters and the residual bending capacity of test specimens were determined after inducing accelerated corrosion. SHCC has a significantly prolonged period of corrosion propagation and maintained the bearing capacity of the beam due to its micro-cracking behavior and ultra-high tensile strain capacity. Test results in [23,24] also showed that SHCC beams possessed better corrosion protection than concrete beams. The excellent performances of SHCC is expected to contribute to improving the sustainability of infrastructure within the service life.

The actual service life of civil engineering structures subjected to combined action can be considerably shorter than that of similar loads but acting separately. Internal damage and cracking of cement-based materials induced by mechanical loads can increase the penetration rate of chloride [25]. If the effect of freeze–thaw action is considered, the deterioration rate will be accelerated. In this paper, the damage characteristics of SHCC specimens under the simultaneous action of flexural loading and freezing and thawing cycles in the chloride environment is demonstrated, which is extremely important for the actual assessment of durability of SHCC in severe conditions.

## 2. Materials and Methods

### 2.1. Material and Specimen Preparation

The materials used in this experiment include 42.5R Portland cement produced by Jidong Cement Co., Ltd., Hohhot, China, class-I fly ash (FA) produced by the Dalad Banner power plant (Ordos, China, microsilica sand (grain size less than 212 microns) (Jiuyuan quartz sand Co., Ltd., Baotou, China), polycarboxylate high-performance water-reducing admixture (Sika Ltd., Dalian, China), ordinary water, and micro polyvinyl alcohol (PVA) fibers. PVA fibers (REC-15) (Kuraray trading Co., Ltd, Osaka) used in the SHCC matrix were produced in Japan, with the following parameters: length 12 mm, diameter 40 microns, tensile strength 1600 MPa, and elastic modulus 40 GPa. In all SHCC specimens, the volume content of PVA fibers was 2%. The proportion of various materials and basic mechanical properties of hardening SHCC are presented in Table 1.

All specimens were cast in a stainless-steel mold and demolded after 24 h. Then, test pieces were moved into the standard curing room with a temperature of 20 ± 2 °C and relative humidity of 95% ± 5% until the testing date. Compressive strength was tested using three 100 mm cube specimens. A dumbbell-shaped specimen with a size of 600 × 330 × 15 mm^3^ was used for the uniaxial tension test; SHCC exhibited a tensile strain capacity of more than 3.0% (uniaxial tension test methods were detailed [26]). Tests of the pore characteristics of hardening SHCC were performed on the Rapid Air 457 system, and results are presented in Table 2. Flexural strength was obtained by testing three 100 × 100 × 400 mm^3^ prism specimens at 28 days, and the result was used to determine the preloading of the freeze–thaw test. After freeze–thaw cycles (100, 200, 300, 400, 500), the residual flexural strength of SHCC specimens was tested using a four-point bending load.

### 2.2. Loading Device and Stress Level

**Figure 1:**
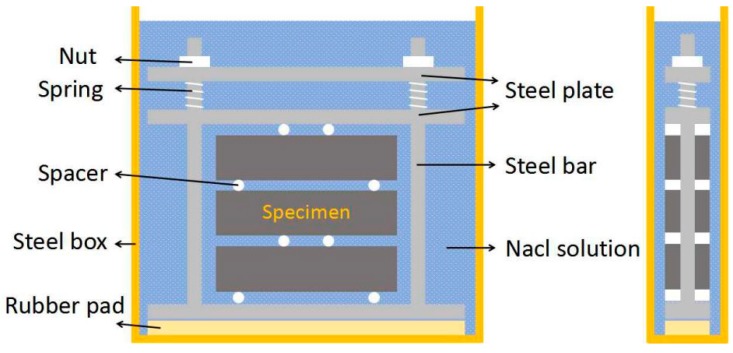
Self-designed loading device for freeze–thaw environment.

The loading device was modified based on the design of [27]. An auxiliary stainless-steel plate was added so that the sustained loading would be more stable in the experiment, and the loading device with steel frame and dual springs is shown in Figure 1. In this study, sustained loading levels corresponding to 0, 36, 54, and 72% of the flexural strength were applied (stress ratio *S* is 0, 0.36, 0.54, 0.72, respectively). Three prism specimens were prepared for each group stress ratio, and the calculation span was 300 mm. According to Hooke’s law, sustained flexural loading was applied by adjusting the spring deformation, and a four-point flexural loading mode was selected. The spring deformation was adjusted to prevent stress relaxation after 100 freeze–thaw cycles or equivalent soaking time. Zinc was used to avoid corrosion of the loading device from the test solution during exposure.

### 2.3. Test Methods

Freezing and thawing cycles tests under the combined action of sustained flexural loading and chloride attack were conducted in accordance with rapid freezing method in the “Standard for Test Methods of Long-Term Performance and Durability of Ordinary Concrete” [28] (GB/T50082-2009, China). The freeze–thaw medium was replaced from freshwater to sodium chloride solution with a mass concentration of 3.5%. Before freeze–thaw cycle tests, loading devices with SHCC specimens were moved into the prefabricated stainless-steel box. Then, the configured sodium chloride solution was injected into the box, and the liquid level height was greater than the loading device. After soaking for four days, the boxes with SHCC specimens were moved into the test chamber for the freeze–thaw cycles test. The test temperature was controlled using a temperature sensor embedded in the center of the sample and ranged from −18 ± 2 °C to 5 ± 2 °C in 2–4 h. SHCC specimens were frozen and thawed in sodium chloride solution.

For each test condition, loss of mass and relative dynamic elasticity modulus (RDEM) were determined through three 100 × 100 × 400 mm^3^ prism specimens every 25 freeze–thaw circles. The test of freeze–thaw cycles was finished when the number of cycles reached the predetermined amount or test specimens were destroyed. The measured results showed that one freeze–thaw cycle took approximately 3 h. To maintain a constant concentration of solution, NaCl solution was refreshed once every 100 freeze–thaw cycles. After 100, 200, 300, 400, and 500 freeze–thaw cycles, surviving SHCC specimens were subjected to a four-point bending load to determine their residual flexural strength. Four-point bending load tests were performed using the Landmark MTS 100 KN servo hydraulic loading system (MTS Systems Corporation, Minneapolis, MN, USA).

Immersion tests under a combination of sustained flexural loading and a chloride environment (without freeze–thaw cycles) were also set up to determine the effect of freeze–thaw action on the flexural strength and chloride diffusion properties in SHCC specimens as a comparative group. In the test of comparative groups, a sustained flexural load level S = 0.36 was also selected. The immersion test and freeze–thaw cycles test were performed simultaneously. When freeze–thaw damage parameters were determined, SHCC specimens from the immersion group were also required to be removed from the chloride solution. All specimens were synchronously moved into the salt solution again after the test was finished.

In the test to determine chloride ion concentration, the pure bending section of cross-sectioned specimens 100 × 100 × 100 mm^3^ was removed with a concrete cutting machine. Part of the 15 mm thickness was removed from the original vertical sides of the specimen. Samples were drilled from the side of the tensile zone to the compression zone side layer-by-layer in the remaining part, and a set of samples was taken every 2 mm. All powder samples were removed using a 0.63 mm sieve. Samples were arranged in aluminum boxes and placed in a 105 ± 5 °C oven for 2 h and cooled to room temperature. According to the “Test Code for Hydraulic Concrete” [29] (SL352-2006, China), chloride ion content at different diffusion depths in SHCC specimens were measured by the Moire method; AgNO_3_ solution was used as the standard solution and K_2_CrO_4_ solution was used as the indicator.

## 3. Results and Discussion

### 3.1. Freezing and Thawing Resistance

#### 3.1.1. Surface Damage and Mass Loss

**Figure 2:**
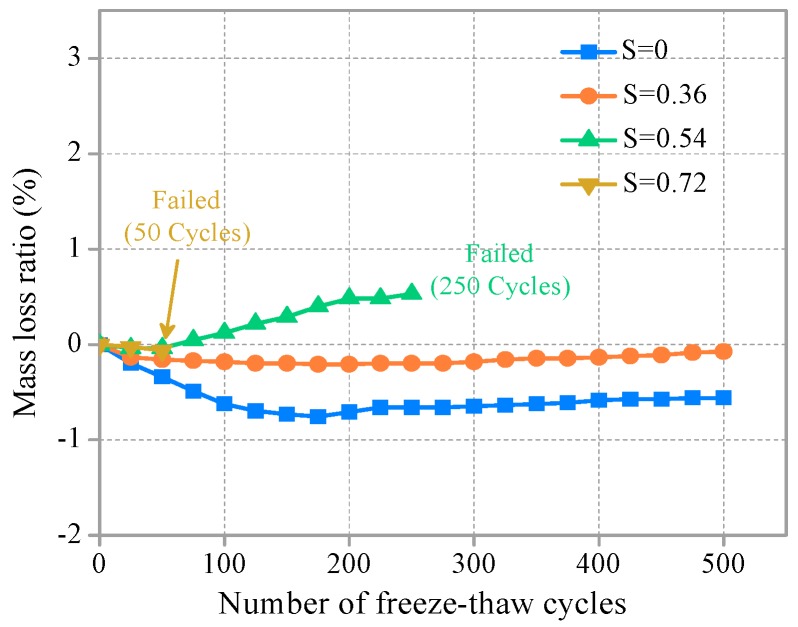
Ratio of mass loss versus number of freeze–thaw cycles.

**Figure 3:**
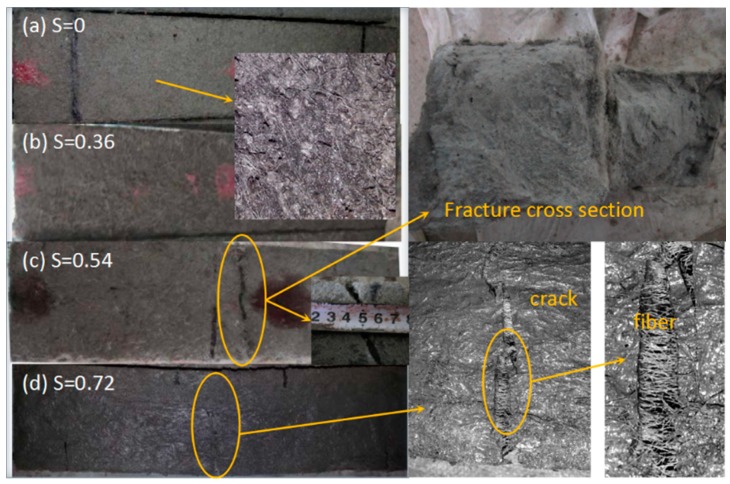
Surface features of SHCC specimens after freeze–thaw cycles. (**a**) S = 0, FT500; (**b**) S = 0.36, FT500; (**c**) S = 0.54, FT250; (**d**) S = 0.72, FT50. FT, freeze–thaw cycles.

Freeze–thaw test results are summarized in Table 3. When the SHCC mass decrease, mass loss rate is positive; when the SHCC mass increase, it is negative. The influence of sustained flexural loading on the frost resistance durability of SHCC specimens was preliminarily assessed through surface damage and mass loss. The relationships between the mass loss ratio and the number of freeze–thaw cycles under different sustained flexural loading levels are shown in Figure 2. The mass of SHCC specimens had no loss after 500 freeze–thaw cycles, but there was a slight increase when S was less than or equal to 0.36. Digital images of SHCC specimens subjected to 500 freeze–thaw cycles at S = 0 and 0.36 are shown in Figure 3a,b, respectively. Surface topography of SHCC specimens showed little damage after 500 freeze–thaw cycles and overall remained intact.

In contrast to S ≤ 0.36, the mass of SHCC specimens showed a slight decreasing trend with an increasing number of freeze–thaw cycles for S = 0.54. The mass loss rate was only 0.53% after 250 freeze–thaw cycles, but the specimen was not destroyed, as shown in Figure 3c. When the stress ratio increased to S = 0.72, fracture failure occurs in SHCC specimens after 50 cycles, as shown in Figure 3d. At that time, the mass loss was only 0.06%.

As can be seen, flexural stress had little effect on the mass loss and surface topography of SHCC specimens under a combination of sustained flexural loading and chloride attack, which is related to the failure mechanism under the action of stress. The concentration of stress in macro and micro cracks caused by freezing and thawing cycles is affected by the applied stress, which accelerates the development of cracks and causes damage and deterioration. Applied stress can also induce micro cracks caused by freeze–thaw cycles that appear in advance. The mass loss in SHCC specimens under different levels of stress was essentially the same. In the initial stage of the hydration reaction, the degree of hydration was higher, and the internal moisture was consumed quickly, with sustained development of hydration reaction. A dense hydrate layer hindered external water so that the degree of hydration was reduced [30]. However, the hydrate layer was destroyed due to freeze–thaw action, so that the mass of the specimen increased, possibly due to further hydration of the activated cementitious materials. In addition, freeze–thaw tests indicated that the addition of polyvinyl alcohol fibers to the SHCC matrix substantially improved frost resistance durability [11,14]. In summary, the mass loss of SHCC specimens was not obvious when subjected to several freeze–thaw cycles under sustained flexural loading and a chloride environment.

#### 3.1.2. Relative Dynamic Elasticity Modulus

**Figure 4:**
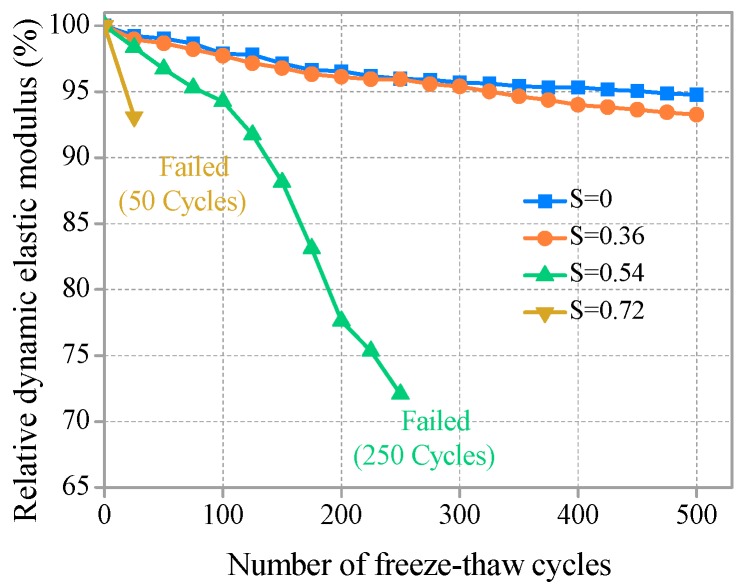
Relative dynamic elastic modulus versus number of freeze–thaw cycles.

To assess the internal damage of SHCC specimens caused by freeze–thaw action, the change in dynamic elasticity modulus was measured. The relationships between relative dynamic elasticity modulus and the number of freeze–thaw cycles for different sustained flexural loading levels are shown in Figure 4. The dynamic elastic modulus decreased with the development of freeze–thaw cycles; S ≤ 0.36, the dynamic elastic modulus, decreased very slightly, and RDEM was greater than 93% after 500 freeze–thaw cycles. Compared to SHCC specimens without sustained flexural load (S = 0), the effect of S = 0.36 on the loss of dynamic elastic modulus is not obvious. The reason may be that the SHCC specimens were in the full elastic stage (S = 0.36), and the internal micro cracks and damage were too slight to accelerate freeze–thaw damage.

The dynamic elastic modulus of SHCC specimens decreased rapidly with the development of freezing and thawing for S = 0.54. In the early stage of freezing and thawing (N < 100), the dynamic elasticity modulus decreased slowly, and it accelerated after 100 cycles. After 250 freeze–thaw cycles, before completing 300 freeze–thaw cycles, SHCC specimens were removed from freeze–thaw tests due to fracture failure. The failure behavior of this type of specimen was sudden fracture. The dynamic elastic modulus of SHCC specimens decreased sharply when the sustained flexural load was increased to S = 0.72, and fracture failure of SHCC specimens was observed after 50 freeze–thaw cycles. Of course, cycles may occur fewer than 50 times (25–50), and the specimens may have been damaged in the freeze–thaw machine. In this case, SHCC showed a sudden brittle failure code. Before fracture failure, the relative dynamic elastic modulus was more than 90%, and suddenly dropped to 0 after failure. Therefore, stress of S = 0.72 played a leading role in the failure of the specimen. However, the effect of relatively low S (S = 0.36) on the damage degradation of SHCC under a combination of freezing-thawing cycles and a chloride environment can be ignored. As shown in Figure 4, the results show that the damage rate of SHCC under the combined action was much larger than for a single freeze–thaw cycle action. Compared to no sustained flexural loading (S = 0), the loss of a dynamic elastic modulus of the SHCC specimens was accelerated after sustained flexural loading (S = 0.36, 0.54, 0.72) was applied. In Figure 4, it can be seen that higher sustained flexural loading led to greater freeze–thaw damage, and the loss of dynamic elastic modulus of the corresponding specimens increased significantly.

The results of [13] showed that compared to the single action of freezing and thawing cycles, the deterioration form of concrete under combined external stress and freezing and thawing action was very different, and damage was accelerated. Compared to the mass loss rate, RDEM can be reasonably reflected by freeze–thaw damage of SHCC specimens under the combined action of sustained flexural loading and the chloride environment. The degree of freeze–thaw damage was S = 0.72 > S = 0.54 > S = 0.36 > S = 0 under an equal number of cycles. Although the freeze–thaw damage to the SHCC specimen was accelerated due to the action of sustained flexural loading, it still showed good frost resistance when S ≤ 0.54. The results of [31] showed that the addition of an appropriate amount of PVA fiber (volume content of 1.5–2%) could effectively strengthen the anti-stripping ability of the SHCC matrix to improve the freezing and thawing resistance of SHCC. Additionally, the air void spacing factor of concrete is one of the most important factors affecting frost resistance [32]. As shown in Table 3, the average spacing factor of SHCC is well below the normal recommended value (200 μm) for excellent frost resistance. From the pore structure parameters in Table 2, good frost resistance for all SHCC specimens can be expected. This excellent performance of SHCC mostly contributed to the void feature of the SHCC matrix, which improved due to the appropriate addition of PVA fibers [11].

#### 3.1.3. Microstructure Characteristics

**Figure 5:**
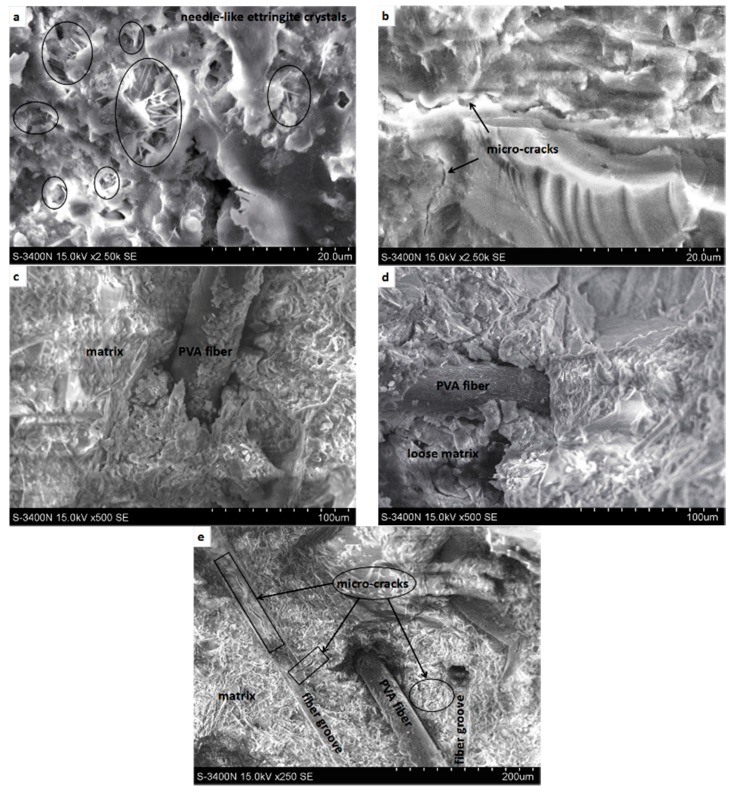
SEM images before and after freeze–thaw cycles. (**a**,**c**) without freeze–thaw cycles; (**b**,**d**,**e**) after 300 freeze–thaw cycles, S = 0.36.

The microstructure and morphology of SHCC were obtained using a scanning electron microscope. Figure 5a presents SEM images of the SHCC sample before freeze–thaw cycles, and needle-like ettringite crystals can be observed in the SHCC matrix. Further hydration of the SHCC matrix continued during the development of freeze–thaw cycles. Needle-like ettringite crystals gradually disappeared and the internal structure of the SHCC matrix became more compact, as shown in Figure 5b. However, the internal structure was damaged by the freezing and thawing action. Micro cracks caused by freezing and thawing action were observed in the SHCC matrix (Figure 5b). The interface bonding between PVA fiber and matrix was in good condition before freeze–thaw cycles (Figure 5c). Compared to the situation without freezing and thawing cycles, the matrix surrounding PVA fiber loosened and cracked after 300 freeze–thaw cycles (Figure 5d). Random longitudinal and transverse micro-cracks (Figure 5e) appeared on the surface of the fiber groove. Figure 5b–e shows that the damage caused by freeze–thaw action was mostly seen as micro cracks. In general, freeze–thaw damage degradation is characterized by the gradual formation of micro cracks in the SHCC matrix.

### 3.2. Residual Flexural Performance

**Figure 6:**
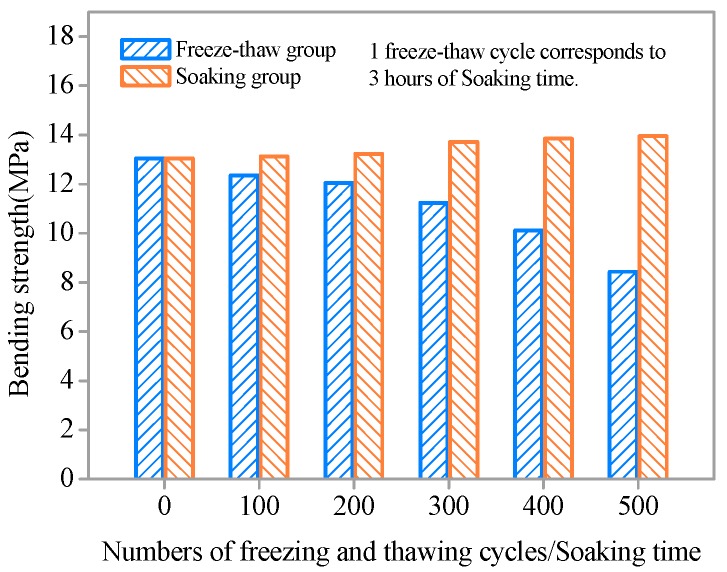
Flexural strength versus number of freeze–thaw cycles/soaking time.

To investigate the effects of freezing and thawing action on the flexural resistance of SHCC specimens under a combined chloride environment and sustained flexural loading (S = 0.36), the residual flexural strength was obtained by four-point bending after 100, 200, 300, 400, and 500 cycles or an equivalent soaking time. Figure 6 shows the results of flexural strength of SHCC specimens subjected to different numbers of freeze–thaw cycles or equivalent soaking time. The flexural strength of all SHCC specimens after freezing-thawing action declined compared to the original specimens and decreased with the development of freezing and thawing. Without freezing and thawing cycles, flexural strength increased with increasing immersion time in a chloride environment. Longer soaking times are correlated with higher flexural strength. However, the growth of flexural strength is not substantial, less than 7%.

**Figure 7:**
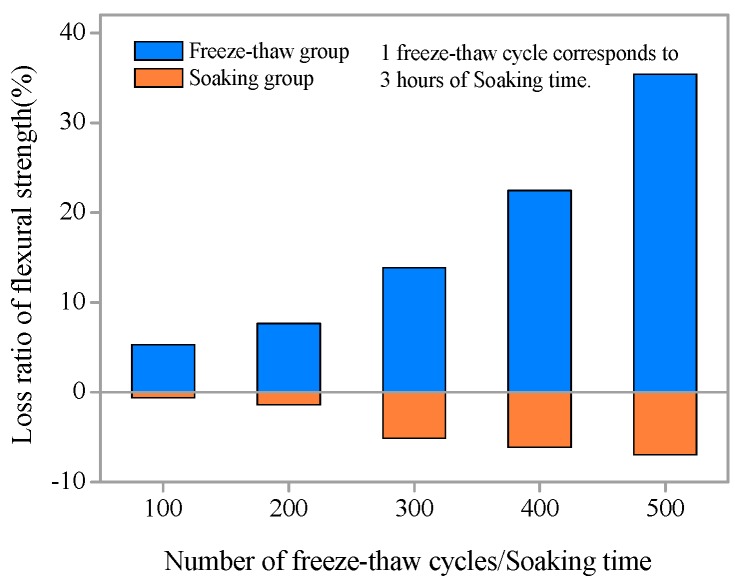
Loss ratio of flexural strength versus number of freeze–thaw cycles/soaking time.

The influence of freezing and thawing action on the flexural strength of SHCC specimens is obvious from Figure 6. With increasing freeze–thaw cycles, the loss ratio of flexural strength of SHCC increases gradually (Figure 7). When the flexural strength of SHCC specimen decrease, the loss ratio of flexural strength is positive; when the flexural strength of SHCC specimen increase, it is negative. After 300 freeze–thaw cycles, the flexural strength of SHCC decreased by only 13.87%. With 500 freeze–thaw cycles, the flexural strength decreased by 35.4%. However, for plain concrete, the loss ratio of flexural strength after the simultaneous actions of both load and freezing-thawing cycles was very serious after a few cycles [33]. Compared to the freezing and thawing group, the loss ratio of flexural strength of SHCC specimens without freeze–thawing was negative, and flexural strength increased gradually with increased soaking time. However, the growth rate was slight, within 7%, as shown in Figure 7.

**Figure 8:**
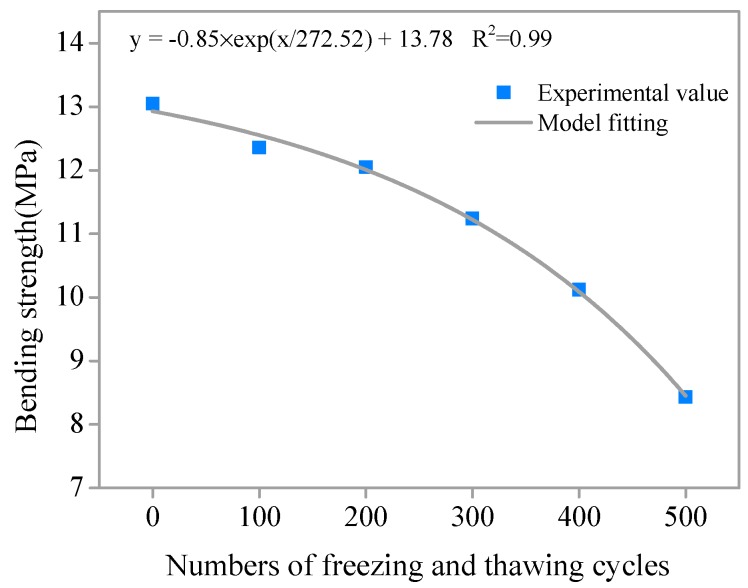
Degradation model of flexural strength after freeze–thaw cycles.

To further clarify the degradation law of the residual flexural strength of SHCC specimens after different numbers of freeze–thaw cycles, a flexural strength degradation model was established. Nonlinear fitting regression of the relationship between residual flexural strength and number of freeze–thaw cycles was performed based on the test results. The residual flexural strength degradation model of SHCC specimens under the coupling action of a salt freezing cycle and flexural loading (S = 0.36) was obtained. As shown in Figure 8, the correlation coefficient *R*^2^ > 0.99, the degradation model and the experimental results are in good agreement. Therefore, this model of the exponential form can be used to predict and describe the degradation of residual flexural strength of SHCC under the coupling action of salt freezing cycles and loading.

### 3.3. Chloride Diffusion Performance

#### 3.3.1. Distribution and Relationship of C*_f_* and C*_t_*

**Figure 9:**
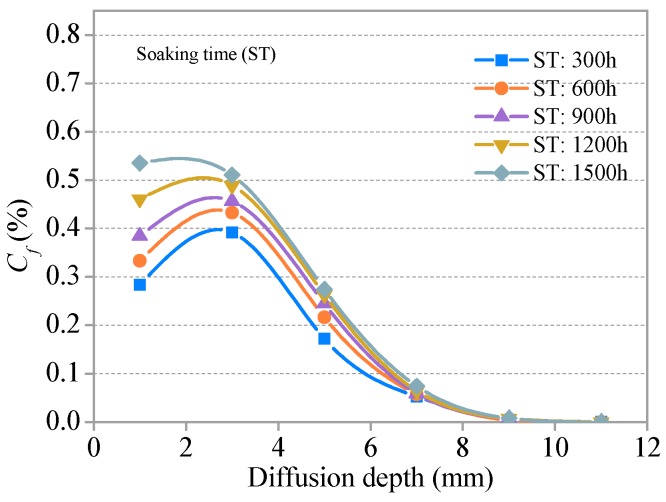
Relationship between C*_f_* and diffusion depth for various soaking times.

**Figure 10:**
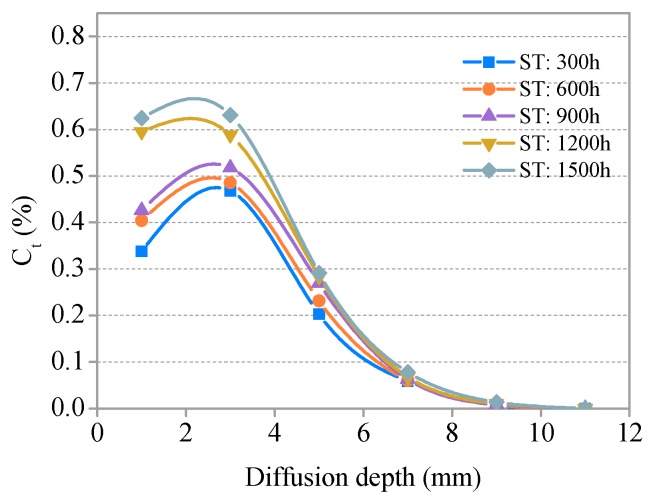
Relationship between *C_t_* and diffusion depth for various soaking times.

The relationships between the free chloride concentration (C*_f_*), total chloride concentration (C*_t_*), and diffusion depth in the SHCC specimens with no freezing and thawing cycle groups (S = 0.36) are presented in Figure 9 and Figure 10, respectively. Distribution curves of C*_f_* and C*_t_* are generally similar in shape. The two curves first increase, then decrease, and finally tend to plateau. There is a peak at a certain depth (0–2 mm) from the diffusion surface of the specimen. C*_f_* and C*_t_* in the same diffusion depth of SHCC specimens increase slightly with increasing soaking time and decrease with increasing diffusion depth in the same soaking time. The effect of soaking time on C*_f_* and C*_t_* of the same diffusion depth is not obvious and is weakened with increased diffusion depth. The C*_f_* and C*_t_* of each group in SHCC specimens is 0.003–0.007% and 0.006–0.014%, respectively, for D > 8 mm, and the influence of soaking time can be neglected.

**Figure 11:**
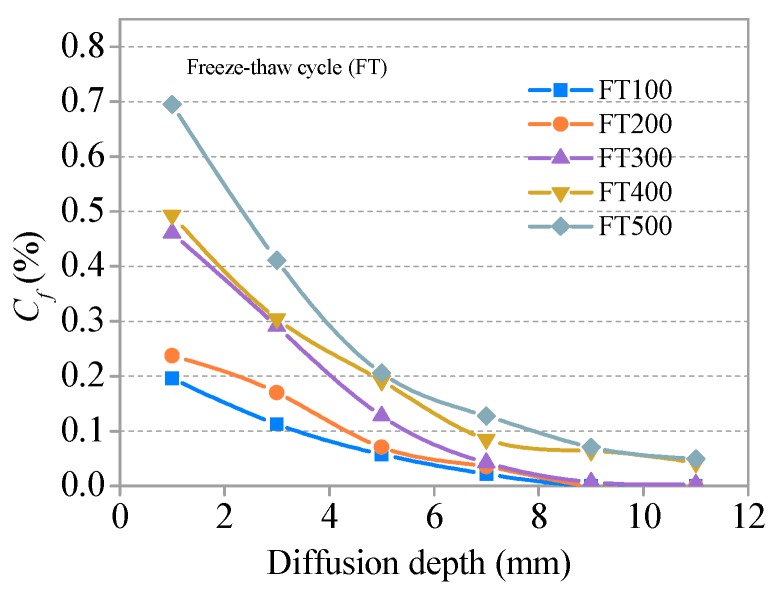
Relationship between *C_f_* and diffusion depth after freeze–thaw cycles.

**Figure 12:**
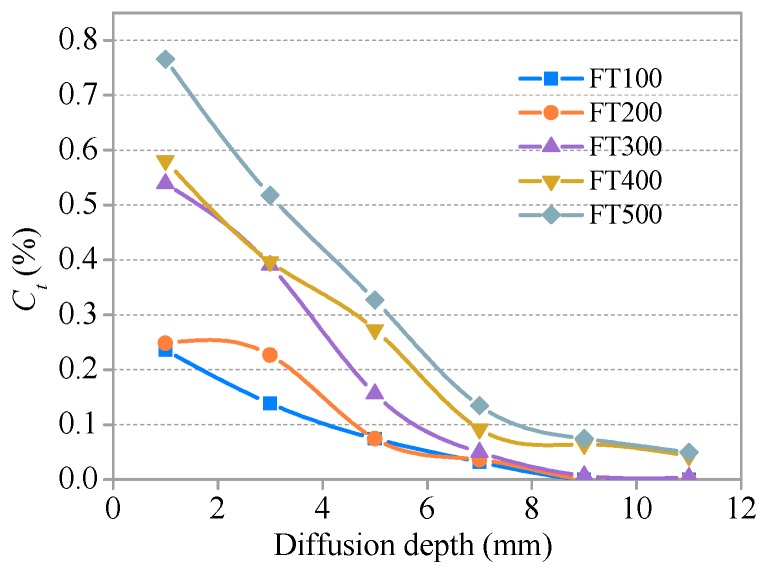
Relationship between *C_t_* and diffusion depth after freeze–thaw cycles.

The relationships between *C_f_* and *C_t_* and the diffusion depth of SHCC specimens after different freeze–thaw cycles (S = 0.36) are presented in Figure 11 and Figure 12, respectively. The development trends for *C_f_* and *C_t_* with a diffusion depth of SHCC specimens under different freeze–thaw damage are basically consistent with the immersion group (without freezing and thawing damage). *C_f_* and *C_t_* increase with the development of freezing and thawing at the same diffusion depth. A comparative analysis of Figure 9, Figure 10, Figure 11 and Figure 12 shows that the effect of freeze–thaw action on the distribution of *C_f_* and *C_t_* in SHCC is significant. The diffusion depth of free chloride ion for specimens under freeze–thaw action is greater than without freezing and thawing cycles. *C_f_* at D = 12 mm for specimens under freeze–thaw action is greater than 0.043% when there are 400 freeze–thaw cycles (or a soaking period) but 0 without freeze–thaw cycles.

**Figure 13:**
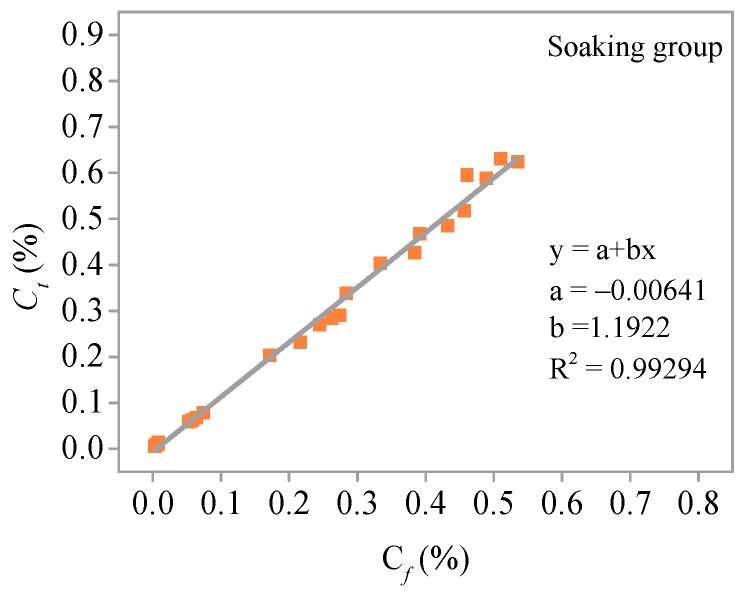
Relationship between *C_f_* and *C_t_* without freeze–thaw cycles.

**Figure 14:**
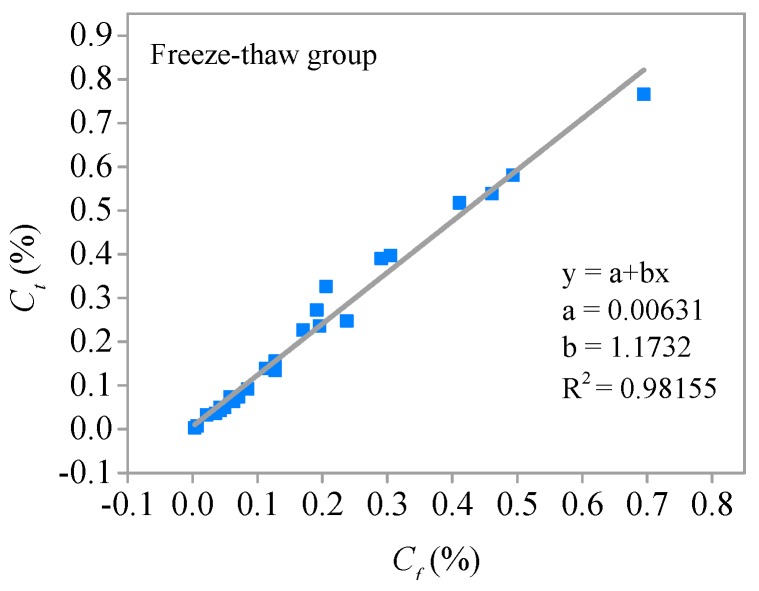
Relationship between *C_f_* and *C_t_* after freeze–thaw cycles.

Linear regression is used to analyze the relationship between *C_f_* and *C_t_* after soaking (without freeze–thaw cycles) and after freezing and thawing cycles, respectively. The relationship between *C_f_* and *C_t_* without and with freezing and thawing is shown in Figure 13 and Figure 14, respectively. *C_t_* increases with increased *C_f_*, and there is a good linear relationship between the two. The correlation coefficient *R*^2^ is 0.99 and 0.98 for the immersion group and the freezing and thawing group, respectively.

#### 3.3.2. Effects of Freeze–thaw Cycles on Total *C_f_* and *C_t_*

**Figure 15:**
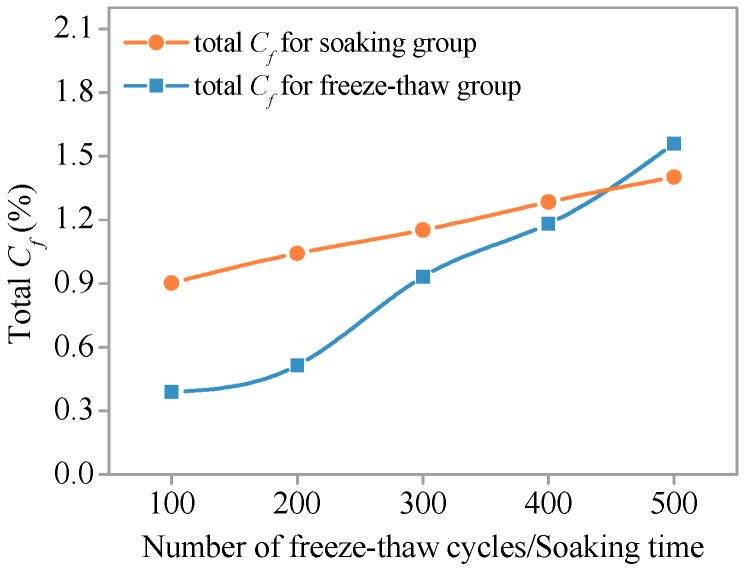
Total *C_f_* versus number of freeze–thaw cycles/soaking time.

**Figure 16:**
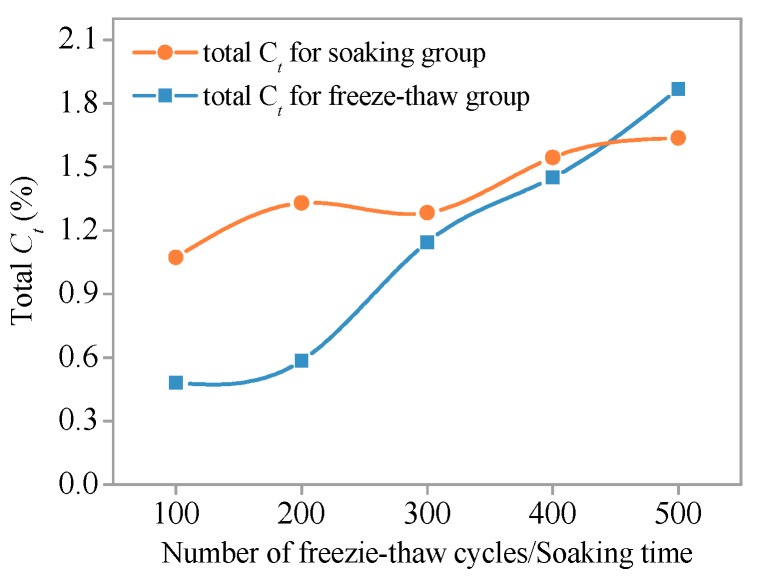
Total *C_t_* versus number of freeze–thaw cycles/soaking time.

Relationships between total *C_f_* and *C_t_* along the diffusion depth (0–11 mm) and number of freeze–thaw cycles (or soaking time) are shown in Figure 15 and Figure 16, respectively. At the early stage, total *C_f_* and *C_t_* of the freeze–thaw group and the soaking group are significantly different. When the number of freeze–thaw cycles is 100 (the corresponding soaking time is 300 h), the total *C_f_* of the soaking group specimen is 2.7-fold higher than the freeze–thaw group specimen, and 2.3-fold higher for total *C_t_*. However, the difference between chloride ion content of soaking group specimen and freeze–thaw group specimen gradually decreased with the increase of freeze–thaw cycles or soaking time. When the number of freeze–thaw cycles (or soaking time) reaches 300, the total *C_f_* of the soaking group is 1.2-fold higher than the freeze–thaw group and that of *C_t_* is 1.1-fold higher. The intersection of concentration curves appears between 400 and 500 freeze–thaw cycles. At this point, the total *C_f_* and *C_t_* of the two groups are equal. Total *C_f_* and *C_t_* of the freeze–thaw cycle group are greater than that of the non-freezing and thawing cycles.

During freezing, chloride migration is hindered by the low-temperature environment and the effect of internal pore water ice. However, the SHCC specimen was damaged due to the effect of freeze–thaw action, and the internal structure gradually becomes loose. In this way, a rich migration space was provided, which led to the increased chloride ion concentration in SHCC specimens subjected to freezing and thawing action. If the freezing and thawing damage is greater, it will contribute to the migration of chloride ions. It is shown that when the number of freeze–thaw cycles is less than 400 and the freeze–thaw damage is smaller, the freeze–thaw cycles may inhibit the migration of chloride ions in SHCC. When freezing and thawing damage reaches a critical value, the freeze–thaw action begins to promote the migration of chloride ions. The critical point is between 400 and 500 cycles, as shown in Figure 15.

#### 3.3.3. Chloride Diffusion Coefficient

Regression analysis was used to fit the nonlinear relationship between *C_f_* and diffusion depth. The fitting function is

*C*(*x*) = *a* × exp(−*x/b*) + *c* (1)

where *x* is the diffusion depth; *C*(*x*) is the diffusion depth of *C_f_* at *x*; and *a*, *b*, *c*, are parameters. The fitting results are shown in Table 4. In addition to 200 cycles, *R*^2^ is greater than 0.98, and there is a higher degree of function fitting. The surface chloride concentration *C*_s_ can be calculated using the function parameters, and the diffusion depth *x* is 0. In this paper, the free chloride concentration *C_f_* in the first layer (0–2 mm) for the immersion group is discarded when the curve is fitted.

The chloride diffusion in SHCC is described by Fick’s second law. The free chloride concentration (or content) of the *x* depth distance SHCC specimen surface is

(2) $$C\left(x,\;t\right)\;=\;C_{s}\left[1\;-\;\mathrm{erf}\left(\frac{x}{2\sqrt{D_{c}t}}\right)\right]$$

where *x* is the diffusion depth distance of the specimen surface (mm), *t* is the exposure time (s), *C*_s_ is the surface chloride concentration (kg/m^3^), *D*_c_ is the chloride diffusion coefficient (m^2^/s), and *C*(*x*,*t*) is the free chloride concentration at depth *x* at time *t* (kg/m^3^).

**Figure 17:**
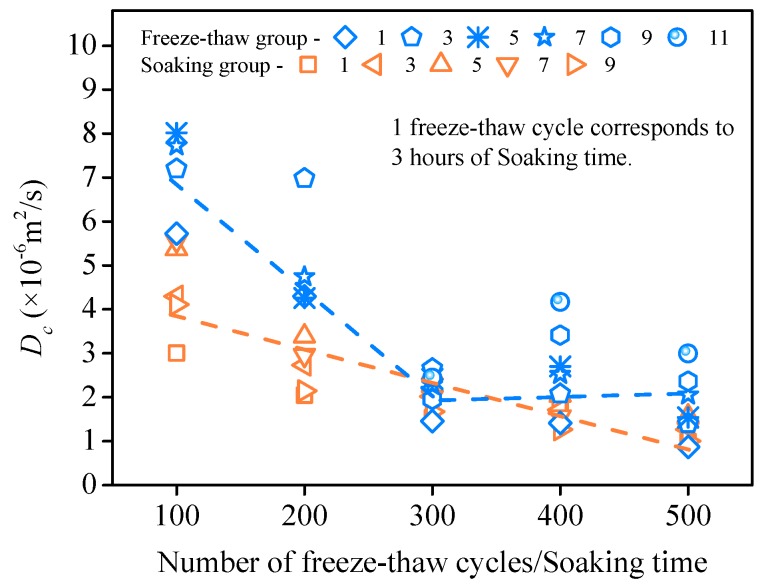
Relationship between *D*_c_ and number of freeze–thaw cycles/soaking time.

*D*_c_ is calculated by Equation (2). MATLAB 7.1 (The MathWorks, Inc., Natick, MA, USA) and the least square method are used in the calculation. The results are shown in Figure 17. For the immersion group, the chloride diffusion coefficient *D_c_* decreases with increasing soaking time. The linear relationship is approximately presented between *D*_c_ and soaking time. The diffusion process for chloride ions is affected by temperature. Lower temperature is correlated with a slower diffusion rate of chloride ions [13]. SHCC proved to have a good frost resistance, and the dynamic elastic modulus decreased slightly after 300 freeze–thaw cycles (see Figure 4). Therefore, low temperature has a great influence on the diffusion coefficient in the early stage of freeze–thaw cycles (<300 times). *D*_c_ decreases with the development of freeze–thaw cycles, as shown in Figure 17. However, the internal damage to SHCC deteriorates with increasing freeze–thaw cycles (>300 times). The dual effect of low temperature and freeze–thaw damage on *D*_c_ tends to be balanced. The turning point appears at approximately 300 freeze–thaw cycles. As shown in Figure 17, *D*_c_ does not decrease with the development of freeze–thaw cycles. However, there is a slight rising trend that is not obvious and may be due to the effect of freeze–thaw damage on the diffusion of chloride ions slightly greater than the inhibitory effect of the low temperature. After this, the effect of freezing and thawing damage on *D*_c_ may play a leading role. A bilinear relationship is found between the chloride diffusion coefficient *D*_c_ and the number of freeze–thaw cycles. Comparative analysis shows that *D*_c_ of the freeze–thaw group is greater than the immersion environment corresponding to the same soaking time. This result may be due to the micro damage caused by freeze–thaw action promoting the diffusion of chloride ions.

## 4. Conclusions

This paper studied the freeze–thaw damage characteristics of SHCC under combined flexural loading at different levels and a chloride environment and analyzed the effect of loading level on frost resistance. Unlike a natural chloride immersion environment, the influence of freeze–thaw action on flexural strength and the chloride diffusion properties of SHCC under the combined load (S = 0.36) were investigated. Using the research in this paper, the following conclusions are drawn:

(1) Four different flexural loading levels had little effect on surface damage and mass loss, and the mass loss of SHCC specimens experienced several freeze–thaw cycles under combined loads from −0.6% to 0.6%. Compared to no flexural loading (S = 0), the reduction of RDEM of SHCC specimens accelerated after sustained flexural loading was applied (S = 0.36, 0.54, 0.72). A higher loading level led to greater freeze–thaw damage, and the RDEM of the corresponding specimen decreased significantly.
(2) With increasing freeze–thaw cycles, the flexural strength of SHCC specimens decreases gradually. After 300 cycles, flexural strength decreased only by 13.87%. The residual flexural strength degradation model of SHCC specimens under the coupling action of salt freezing cycles and flexural loading (S = 0.36) was obtained using nonlinear fitting regression.
(3) *C_f_* and *C_t_* increase with the development of freezing and thawing at the same diffusion depth, and a bilinear relationship was found between the chloride diffusion coefficient *D*_c_ and the number of freeze–thaw cycles. Comparative analysis shows that the *D*_c_ of the freeze–thaw cycle group was greater than for the immersion environment corresponding to the same soaking time.

## Figures and Tables

**Table 1:** Mixture proportions of SHCC matrix and the basic mechanical properties of hardened SHCC.

Components/Property	Unit	Measurement
Cement	kg/m^3^	745
Fly ash		319
Water		319
Silica sand		639
Water-RAE		30
PVA fiber		26
Compressive strength, 28d	MPa	56
Tensile strain, 28d	-	0.034
Tensile strength, 28d	MPa	5.2
Flexural strength, 28d	MPa	13.05

**Table 2:** Properties of the pore structure of SHCC.

Specimen	No. 1	No. 2	No. 3	Mean
Air content (%)	1.35	1.03	1.18	1.19
Specific surface (mm^−1^)	50.33	58.77	58.11	55.74
Spacing factor (mm)	0.176	0.169	0.203	0.183
Void frequency (mm^−1^)	0.170	0.151	0.098	0.140
Average chord length (mm)	0.079	0.068	0.069	0.072

Note: Only chords from 30 to 4000 microns are included in results.

**Table 3:** Freeze–thaw damage characteristics.

Measure	S = 0	S = 0.36	S = 0.54	S = 0.72
No. of cycles completed	500	500	250	50
Mass loss ratio (%)	–0.56	–0.07	0.53	–0.06
Relative dynamic elasticity modulus	94.77	93.26	72.09	0

**Table 4:** Nonlinear fitting results.

No.	FT100	FT200	FT300	FT400	FT500	ST300	ST600	ST900	ST1200	ST1500
a	0.296	0.520	0.677	0.645	0.928	0.008	1.239	1.253	1.346	1.402
b	4.649	10.017	4.038	4.075	3.449	0.058	3.639	3.943	4.113	4.558
c	–0.043	–0.229	–0.059	–0.009	0.005	0.006	–0.108	–0.130	–0.155	–0.200
*R* ^2^	0.999	0.943	0.984	0.992	0.996	0.999	0.998	0.995	0.995	0.989
*C*_s_	0.253	0.291	0.619	0.406	0.933	1.206	1.131	1.123	1.191	1.221

FT, freeze–thaw cycles; ST, soaking time corresponding to freeze–thaw cycles.
